# Zoon’s Vulvitis and Coexisting Lichen Planus: A Rare Vulval Dermatosis

**DOI:** 10.7759/cureus.74896

**Published:** 2024-12-01

**Authors:** Tayyiba Nasreen, Marina O'Kane

**Affiliations:** 1 Department of Dermatology, James Connolly Hospital Blanchardstown, Dublin, IRL; 2 Department of Dermatology, Beaumont Hospital, Dublin, IRL

**Keywords:** coexisting lichen planus, histopathology (hp), plasma cell vulvitis, rare skin disease, risk of malignancy

## Abstract

This case report describes Zoon’s vulvitis or plasma cell vulvitis (PCV) with coexisting lichen planus (LP) treated with methotrexate. PCV is a rare, chronic, benign idiopathic inflammatory condition of the vulvar mucosa, characterized by a bright-red, chronic lesion of mucosa. Typically, it presents as atrophic, shiny, red plaques that can affect any part of the vulva and can spread symmetrically and bilaterally with the propensity of chronicity and gradual coalescence. LP is an uncommon inflammatory dermatosis that can affect skin, nails, and mucosa. There is a strong association between vulvovaginal and oral LP. Clinical presentation is similar to PCV, however, LP has the potential for malignant transformation between 0 % and 5.6%. Our patient was a 60-year-old diabetic female who had a nine-month history of progressive vulvar irritation and itch associated with dyspareunia, urinary stream disturbance, and occasional mouth soreness. Pelvic examination revealed well-demarcated red-orange areas of erythema covering the clitoral hood with effacement, obvious peri-urethral scarring, and fusion of the labia. Oral buccal mucosa revealed bilateral buccal mucosal lichenoid changes with erosive gums in addition to white stria or Wickham stria classic of LP. The potent topical steroid, Clobetasol propionate, provided relief initially, but the patient became intolerant following long-term use and stayed non-respondent, which prompted the vulval mucosal skin biopsy. Histology showed erosion of the surface epithelium with a dense band-like infiltrate of plasma cells in the underlying sub-epithelium with lymphocytes and neutrophils. The presence of 90% plasma cell-rich lichenoid inflammatory infiltrates with less than 1% of lymphocytes and neutrophils. The clinicopathological diagnosis was suggestive of PCV with coexisting LP. There is convincing evidence from studies that early and accurate diagnosis is important, as, unlike Zoon's balanitis, PCV is benign. However, the coexisting LP makes it necessary for regular dermatology follow-up.

## Introduction

Zoon’s vulvitis, vulvitis chronic plasma cellularis or plasma cell vulvitis (PCV) is a rare, chronic, benign idiopathic inflammatory condition of the vulva, characterized by a bright-red lesion of mucosa with significant chronicity. Typically, it presents as thinning of the epidermis of mucosa or atrophic mucosa with shiny, orange-red plaques, which can affect any part of the vulva and can spread symmetrically and bilaterally with the propensity of chronicity and gradual coalescence. It can involve the oral cavity, lips, or palate. Patients with coexisting autoimmune diseases have been documented to experience PCV, indicating an autoimmune etiology [1]. Histologically, it is called PCV due to the increased number of chronic inflammatory responses, such as plasma cells, observed in the skin biopsy. There may be fewer eosinophils and neutrophils. It appears as a dense, subepithelial mononuclear cell infiltrate largely composed of plasma cells, along with diamond-shaped keratinocytes, lozenge-shaped cells, hemosiderin deposition, and red cell extravasation [2]. Zoon's vulvitis is an under-recognized condition and diagnosis is often delayed and can be refractory to topical treatment. The diagnosis is important because symptoms of PCV may improve with topical therapy but signs of the disease can be quite refractory causing a significant impact on the patient‘s quality of life. [1]

Lichen Planus (LP) is an uncommon inflammatory dermatosis, which can affect skin, nails, and mucosa. Approximately 10% of those affected have LP of the nails while half of those affected have oral lichen planus (OLP), which is more common in women than in men. The mucosal LP has more chronicity in its course than the cutaneous LP. It has a strong association between vulvovaginal and oral type [3]. Clinical presentation is similar to PCV, usually characterized by pain, burning, or itching, and associated with dysuria, dyspareunia, and postcoital bleeding. However, the potential for malignant transformation in LP ranges from 0% to 5.6%, which underscores the importance of its early diagnosis and treatment [4]. Female genital LP has four clinical forms, including erosive, papulosquamous, and hypertrophic, which is rare. Diagnosis is clinical in classic cases with white interlacing stria called Wickham stria. However, a biopsy is needed for atypical presentation. The exact cause of OLP is not fully understood, but lymphocytic infiltration suggests that OLP may be a cell-mediated immune response or an autoimmune reaction targeting specific skin cells called keratinocytes. Histology shows hyperkeratosis, saw tooth acanthosis, wedge shape hypergranulosis, and lymphohistiocytic infiltrate obscuring dermo-epidermal junction are classic of mucosal LP [4-7].

## Case presentation

**Figure 1:**
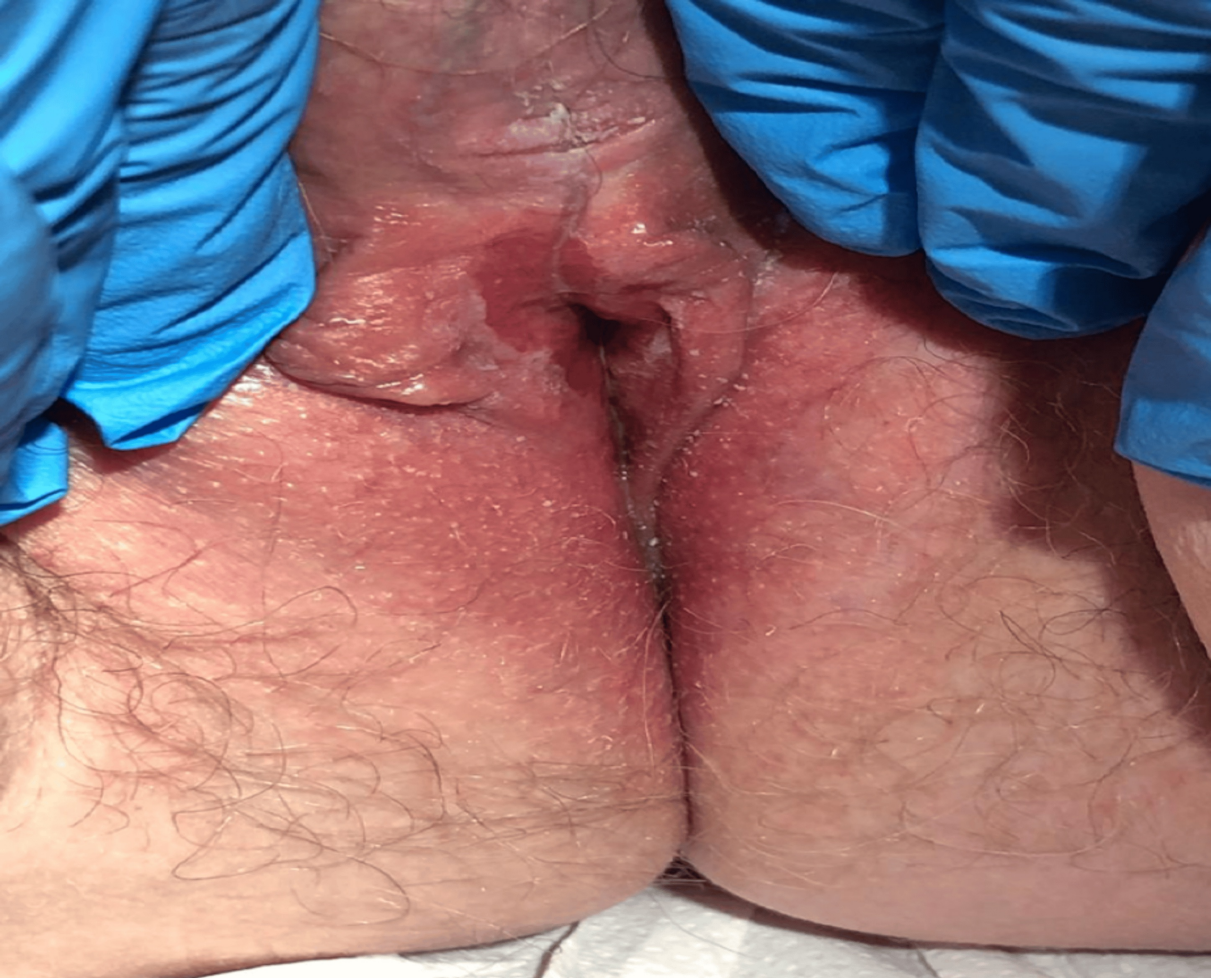
Pelvic examination revealed well-demarcated red-orange areas of erythema covering the clitoral hood with effacement, obvious peri-urethral scarring, and fusion of the labia

A 60-year-old diabetic female had a nine-month history of progressive vulvar irritation and itching, associated with dyspareunia, urinary stream disturbance, and occasional mouth soreness, significantly affecting her quality of life. Pelvic examination revealed well-demarcated red-orange areas of erythema covering the clitoral hood with effacement, obvious peri-urethral scarring, and fusion of the labia as shown in (Figure 1)

**Figure 2:**
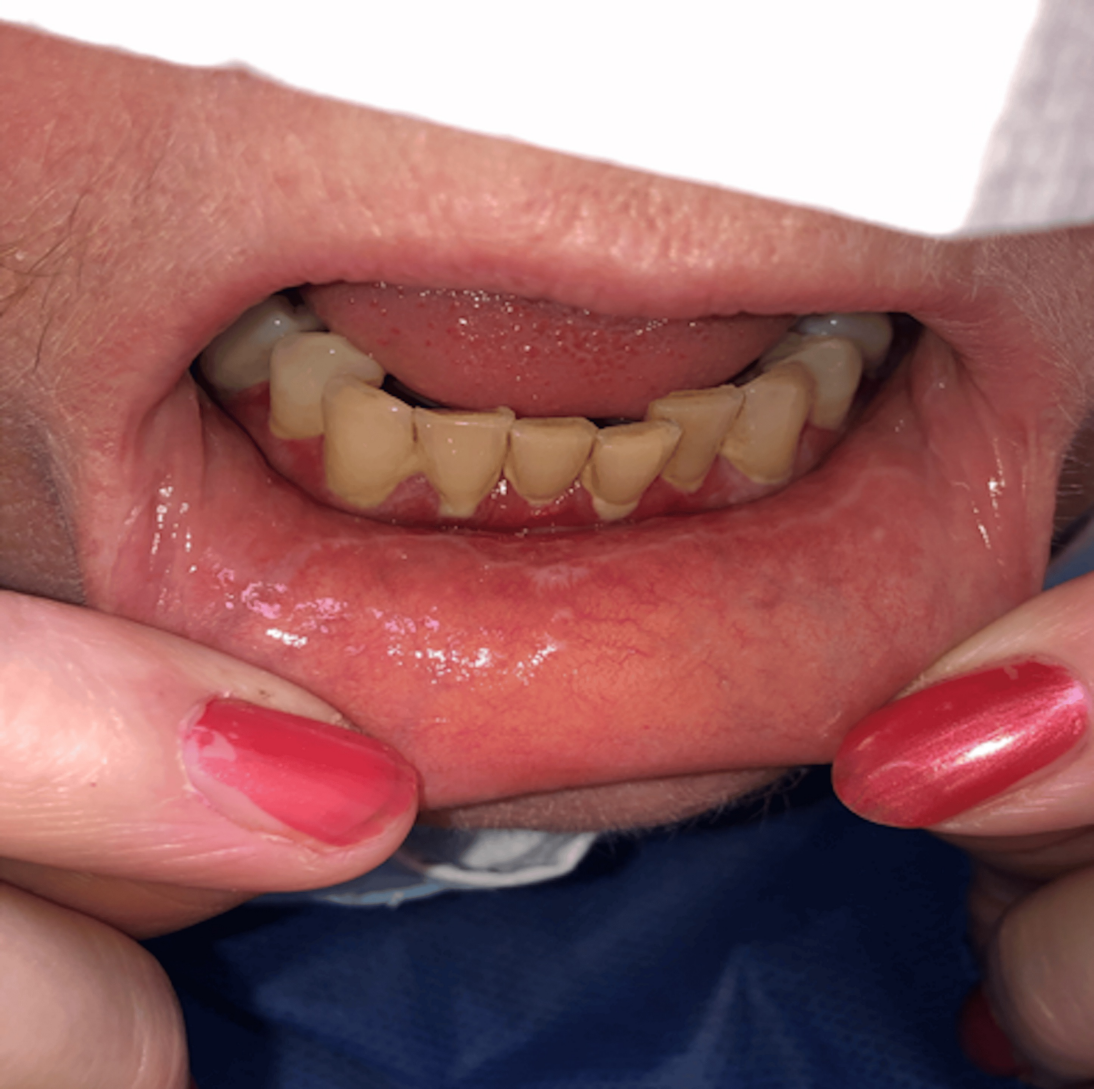
Plasma cell gingivitis: sharply demarcated, erythematous, oedematous gingivitis extending to the mucogingival junction, with some degree of lichenoid changes

**Figure 3:**
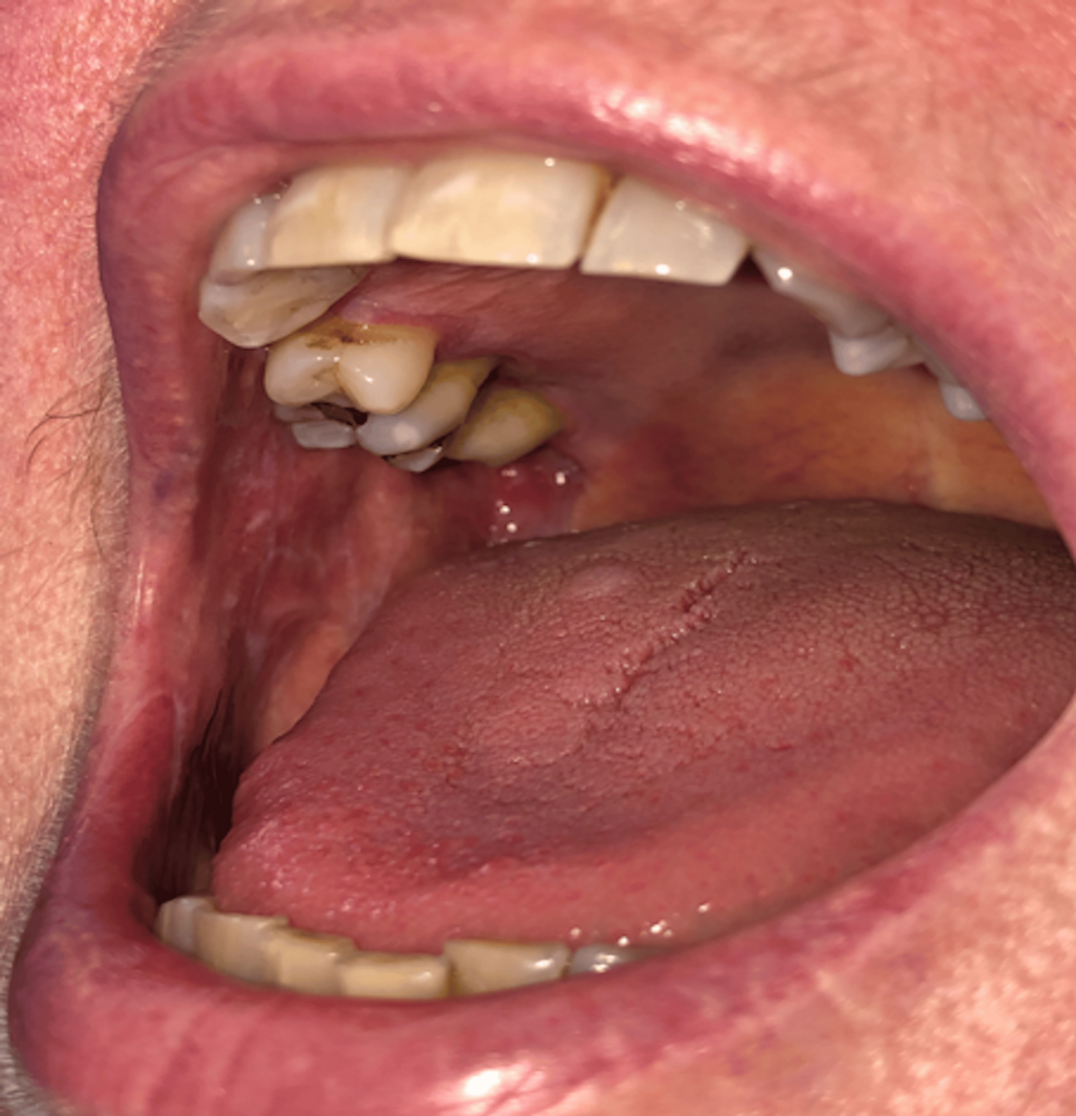
Oral buccal mucosa revealed classic bilateral buccal mucosal lichenoid changes, with erosive gums

Oral buccal mucosa revealed classic bilateral buccal mucosal lichenoid changes with erosive gums. The Wickham stria was classical of LP as shown in (Figures 2, 3). Investigations including FBC, ESR, LFTs, TSH, ANA, and Hep C serology were all unremarkable.

Hair, nails, and rest of full skin examination were normal. There had been no relief with topical antifungal creams, antibiotics, or estrogen suppositories. She found topical tacrolimus stingy and could not tolerate it. The potent topical steroid, Clobetasol propionate, provided relief initially, but the patient became intolerant following long-term use and stayed non-respondent, which prompted the skin biopsy.

**Figure 4:**
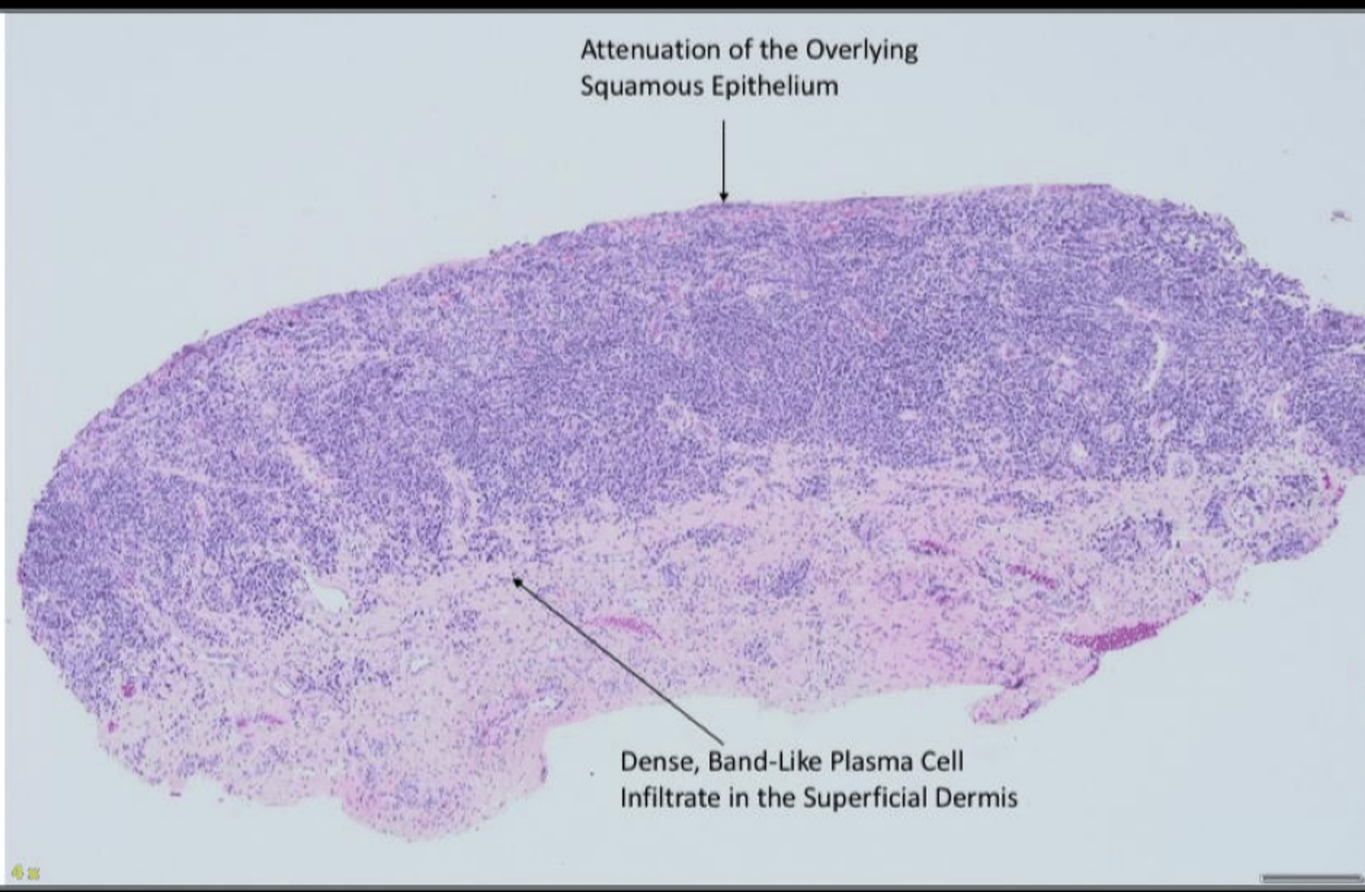
A 4 mm punch biopsy from the right clitoral site Attenuation of the superficial squamous epithelium in the epidermis, with a dense band-like plasma cell infiltrate in the superficial dermis

**Figure 5:**
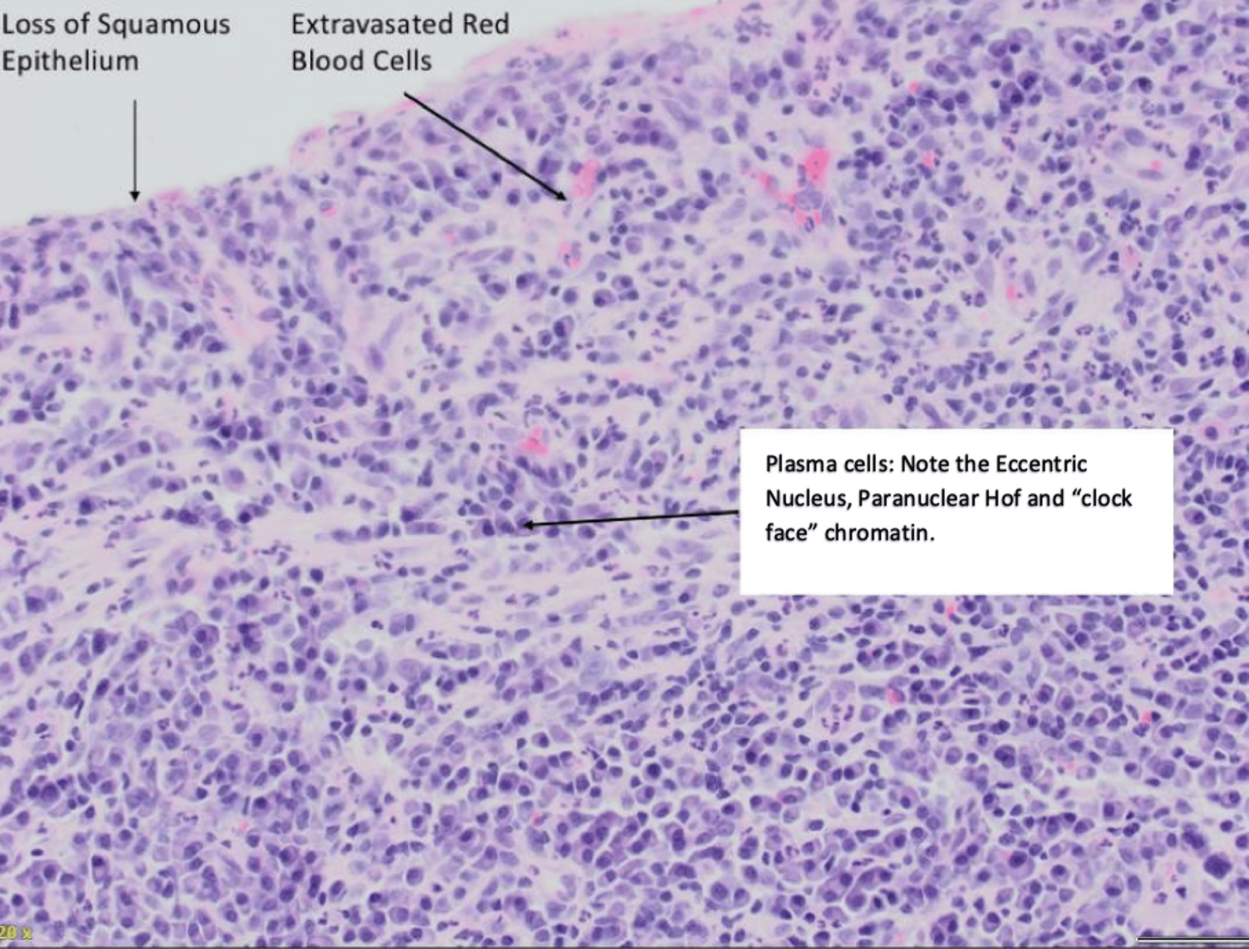
20X H&E: loss of squamous epithelium with extravasation of red blood cells, whereas plasma cells have an eccentric nucleus and paranuclear chromatin

A 4 mm punch biopsy, with the patient's consent, was taken from the right clitoral site, as demonstrated. The biopsy showed erosion of the surface epithelium with a dense band-like infiltrate of plasma cells in the underlying sub-epithelium, along with lymphocytes and neutrophils, as shown in Figures 4, 5. The presence of 90% plasma cell-rich lichenoid inflammatory infiltrates, with less than 1% of lymphocytes and neutrophils, was suggestive of PCV. Periodic acid-Schiff (PAS) staining was negative for fungi. The clinico-pathological diagnosis was suggestive of Zoon's vulvitis and coexisting reticular LP, due to the clinical features of interlacing white lines (Wickham striae).

As patient was non-respondent to topical steroids and the addition of coexisting LP directed toward systemic treatment options. She was offered methotrexate for long-term treatment with dermatology follow-up. Her symptoms started improving after three months, and at the six-month follow-up visit, her symptoms had completely settled, and the clinical signs showed minimal inflammation. She was continued on methotrexate with blood monitoring of LFTs and FBC to keep her disease under control.

## Discussion

Any type of lichenoid dermatosis raises the chance of developing another one. Our patient, in addition to PCV, had coexisting LP, as evidenced by the white interlacing striae of Wickham and its correlation with histological findings. It is anticipated that 2% of women will develop LP, with the oral cavity being the most typically affected. In postmenopausal women, vulvovaginal LP accounts for 6% of chronic vaginal complaints, affects 25% to 57% of OLP cases and is histologically verified in 3.7% of cases involving women who visit a multidisciplinary vulvar clinic [5].

The percentage of plasma cells appears to be the most crucial factor in PCV diagnosis, and when plasma cell counts are ≥50%, this is sufficient for the diagnosis. Numerous plasma cells are often observed in conditions such as eroded contact dermatitis of the genital area, as well as in infections like syphilis or HIV. Special stains and serological testing can be valuable when there is clinical suspicion of an infectious etiology. The presence of a band-like lymphocytic infiltration is considered a key histopathological feature in the pathogenesis of OLP. Additionally, the involvement of B cells in the pathogenesis of OLP is supported by findings from Mattila et al. who reported the presence of B cells in 74.3% of OLP lesions, further highlighting their potential role in the disease process [1,2,7,8].

Chan and Zimarowski et al. describe that basal keratinocyte crowding was a novel finding in their study. This should be considered in future histopathologic investigations since it could be a valuable criterion for identifying PCV in the future (Table 1).

**Table 1:** Key histological features of Zoon's vulvitis and LP LP: Lichen planus

	Histological features
Zoon’s vulvitis	dense lichenoid infiltrate in theupper and middle dermis is largely composed of plasma cells (>50%). Additional findings include vascular proliferation, hemosiderin deposits, and extravasation of erythrocytes
LP	Irregular epidermal hyperplasia, resulting in a characteristic saw-tooth appearance, accompanied by wedge-shaped hypergranulosis. Lymphocytic infiltrate, forming a dense band within the superficial dermis. The basal epidermal layer shows vacuolar degeneration, with marked necrosis of individual keratinocytes

There is no gold standard treatment for PCV; however, the primary treatment is topical corticosteroid, tacrolimus, or topical estrogen. Patients are often refractory to its primary treatment, which significantly affects their quality of life, as reflected in our case, where PCV was recalcitrant to topical potent steroids and tacrolimus. Other alternative therapies are topical imiquimod, estrogen, interferon, lasers, cryotherapy, intralesional corticosteroids, and surgical excision. Systemic treatments include methotrexate and ciclosporins [1,7,9].

Another alternative treatment to recalcitrant PCV demonstrated by Paras Oil et al. is platelet-rich plasma, with significant improvement after two weeks of the first session and complete resolution within six weeks (Table 2) [3].

**Table 2:** Differential diagnosis and treatment options for Zoon's vulvitis and LP LP: Lichen planus

	Differential diagnosis	Treatment options
Zoon‘s vulvitis	Syphilis, HIV, Paget's disease, mucosal LP, genital erosive contact dermatitis, erythroplasia of Queyrat, and pemphigus vulgaris	Topical corticosteroids, calcineurin inhibitors, estrogen, antifungals. Interferon, laser, and cryotherapy
LP	Leukoplakia, candidiasis, lichen sclerosus, lupus erythematosus, chronic ulcerative stomatitis, squamous cell carcinoma, oral manifestation of chronic graft-versus-host disease, and oral lichenoid hypersensitivity	Topical corticosteroids, calcineurin inhibitors, topical retinoids, intralesional injections, systemic treatment with hydroxychloroquine, and methotrexate

Nevertheless, these topical medicines do not consistently provide an effective treatment response. One known fact in treating PVC is limited knowledge of vaginal absorption of the drug, as it is dependent on penetration across the membrane and the solubility of the drug in the vaginal lumen. Vaginal epithelium thickness, mucus viscosity, the pH and volume of vaginal fluid-all of which vary from patient to patient, influencing individual drug absorption. Medications meant to be delivered vaginally must be somewhat soluble in water. Additionally, compared to large-molecular weight lipophilic or hydrophilic medications like testosterone and hydrocortisone, low-molecular-weight lipophilic options like progesterone and estrone are absorbed through the vagina. The suppository form of hydrocortisone still prolongs the drug's contact with the vaginal epithelium to enhance hydrocortisone's low absorption [10].

There is a low safety risk profile with local estrogen therapy with estradiol vaginal tablets as studies have not demonstrated an increased risk of endometrial cancer, breast cancer, or cardiovascular events. Vaginal cream containing 0.01% estradiol is also well tolerated; however, compared to low-dose vaginal pills, the cream absorbs more systemically. Dermatologists should talk about treatment with their patients' oncologists if their patients are receiving treatment for breast cancer or have a history of breast cancer [11,12].

Despite having very distinct clinical symptoms, PCV is a rare inflammatory vulvar dermatosis, as healthcare providers are not familiar with it, diagnosis is sometimes delayed [1]. Symptoms of PCV are common and often quite severe. The frequent delays in diagnosis and the use of inappropriate treatments suggest that misdiagnosis is a common issue. Correct diagnosis is important as the presentation mimics other genital conditions such as Bowen’s disease squamous cell carcinoma, candidiasis, syphilis, herpes simplex, bullous disorders, and extramammary Paget’s disease, which require specific treatment [13,14]. Similarly, it is essential to differentiate LP from erosive LP and vulval intraepithelial neoplasia histologically.

While examples of lesions with moderate dysplasia have been reported, there have been no reports of malignant alterations associated with Zoon's vulvitis, in contrast to Zoon balanitis and LP. Therefore, in order to rule out human papillomavirus (HPV) infection or other dysplastic conditions such as neoplasia and bullous disorders, patients are advised to have periodic gynecologic evaluations, repeat biopsies of persistent lesions, and regular dermatological follow-ups [2]. In addition, variables related to infections, hormones, and irritation have been linked to it.

To the best of our knowledge, very few cases of coexisting PCV and lichen sclerosus have been reported in the literature, and none to date have involved LP, making our case exceptionally rare [7].

## Conclusions

The above case expands our knowledge of challenging clinical and histological diagnoses of two conditions together. Due to the disease's rarity and its differential diagnosis, when discussing treatment options with patients, the multitude of clinical manifestations must be carefully considered. Our patient received methotrexate, and optimum resolution was achieved within three months. Early and correct diagnosis is important as unlike Zoon‘s Balanitis, PCV is benign but coexisting LP makes it liable for regular dermatology follow-up and monitoring of methotrexate side effects.
